# Pan-cancer analysis identifies *ITIH1* as a novel prognostic indicator for hepatocellular carcinoma

**DOI:** 10.18632/aging.202765

**Published:** 2021-03-21

**Authors:** Qing-hua Chang, Ting Mao, Yan Tao, Tao Dong, Xuan-xuan Tang, Guo-hong Ge, Zi-jun Xu

**Affiliations:** 1 Department of Internal Medicine, The Affiliated Third Hospital of Jiangsu University, Zhenjiang, China; 2 Department of Radiology, The Affiliated Third Hospital of Jiangsu University, Zhenjiang, China; 3 Department of Hepatology, The Affiliated Third Hospital of Jiangsu University, Zhenjiang, China; 4 Laboratory Center, Affiliated People’s Hospital of Jiangsu University, Zhenjiang, China

**Keywords:** inter-α-trypsin inhibitor heavy chain (ITIH) gene family, gene expression, pan-cancer, prognosis, liver hepatocellular carcinoma

## Abstract

Although a previous pan-cancer study has reported the expression patterns of *ITIHs* in various tumors, their analyses have been restricted to limited cancer types. We thus comprehensively analyzed the expression profiles and clinical significances of *ITIHs* in a broader spectrum of cancers from TCGA. Our results showed that *ITIHs* were primarily down-regulated in tested cancers. The *ITIH* members were associated with either survival advantage or disadvantage, depending on the cancer type tested and the genes queried. Importantly, we for the first time demonstrated that *ITIH1* had substantially decreased expression in liver hepatocellular carcinoma (LIHC) compared with corresponding normal tissue, and its down-regulation adversely impacted patient outcome. Moreover, *ITIH1* expression was consistently declining during the progression of LIHC. Further analysis revealed that *ITIH1* may be involved in cellular metabolic processes. Our findings established *ITIH1* as a potential diagnostic and prognostic biomarker for LIHC, which awaits future experimental validation.

## INTRODUCTION

The inter-α-trypsin inhibitor (*ITI*) family is a group of protease inhibitors containing one light chain (bikunin) and five heavy chains (*ITIHs*) [1]. *ITIHs* consist mainly of five members: *ITIH1*, *ITIH2*, *ITIH3*, *ITIH4*, and the newly discovered *ITIH5*; they were encoded by five distinct genes located on two different chromosomes-chromosome 3p2.11-12 (*ITIH1*, *ITIH3*, and *ITIH4*) and chromosome 10p14-15 (*ITIH2* and *ITIH5*) [2, 3]. All *ITIHs* are robustly expressed in the liver tissue except for *ITIH5*, which is predominantly expressed in female reproductive tissues [3, 4]. The main function of *ITIH* proteins is to stabilize the extracellular matrices by binding hyaluronic acid (HA) [5]. Since two hallmarks of metastatic tumors are extracellular matrix degradation and activation of the epithelial-to-mesenchymal transition (EMT) program [6], it is reasonable to hypothesize that *ITIHs* may have an anti-metastatic effect in cancer progression. Indeed, an *in vivo* experiment has already shown that the number of tumor metastases significantly decreased upon *ITIH1* and *ITIH3* overexpression [7]. Also, the *ITIH* genes were found to be significantly down-regulated in multiple human solid tumors as reported by a previous pan-cancer study [4]. These suggest that *ITIH* family genes may play a pivotal tumor suppressor role that deserves further investigation. However, progress on the role of *ITIHs* in specific tumors is still limited. For example, *ITIH5* has been reported to be significantly down-regulated and hyper-methylated in breast cancer (BC), and high *ITIH5* expression was associated with favorable outcomes in BC patients [8]. Another member, *ITIH4*, has been demonstrated as a potential diagnostic marker in hepatocellular carcinoma (HCC) that was even superior to AFP; it exhibited a strikingly lower concentration in HCC than normal controls and its expression level was declining during the progression of HCC [9]. Despite that *ITIH1*-*ITIH4* were all predominantly expressed in the liver, whether *ITIH1*-*ITIH3* were similarly dysregulated in HCC is largely unknown.

In addition to the few cancer-related studies, *ITIHs* have also been reported to be involved in inflammatory diseases, such as rheumatoid arthritis and inflammatory bowel disease, and *ITIH4* was shown to be an anti-inflammatory marker-protein in acute ischemic stroke [2, 10]. These findings suggest that *ITIH* proteins may play vital roles in immune responses. Therefore it would also be of interest to study the relationship between *ITIHs* expression and the tumor immune microenvironments.

Previous pan-cancer study on the *ITIH* family has only explored the expression patterns of *ITIHs* in 13 cancer types with relatively small sample sizes, and it has been mostly concerned with the expression and clinical significance of *ITIH2* in breast cancer [4]. Therefore, in this study, drawing on the high-throughput sequencing data from TCGA project, we comprehensively evaluated transcriptional levels and prognostic significances of *ITIH* members with an enlarged sample size and a broader spectrum of cancer types. Importantly, we for the first time demonstrated *ITIH1* as a gene significantly down-regulated in HCC and its down-regulation was associated with worse outcomes in HCC patients; these findings were confirmed in multiple independent datasets. We also explored the genomic alterations of *ITIH1* and the associations of *ITIH1* expression with tumor immune activities across different cancer types. In addition, we studied the gene expression signatures associated with *ITIH1* in order to gain further insight into the biologic importance of *ITIH1* expression in cancers.

## RESULTS

### Expression patterns of ITIHs in normal tissues and cell lines

**Figure 1:**
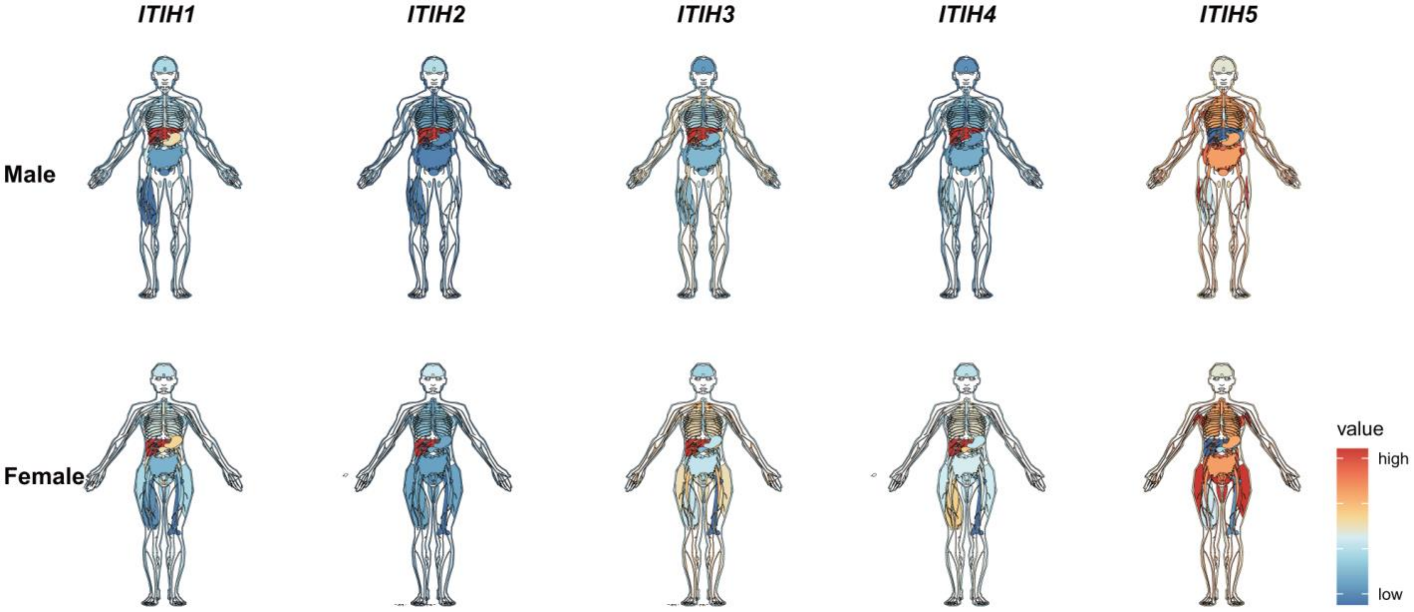
mRNA expression levels of ITIHs in normal tissues of male and female from the Genotype-Tissue Expression (GTEx) database.

Using RNA-sequencing data from the Genotype-Tissue Expression (GTEx) project, we analyzed the expression pattern of *ITIHs* in different tissues from healthy people. We found a preferential enrichment of *ITIHs* in the liver tissue with the exception of *ITIH5*, which, on the contrary, exhibited the lowest expression in the liver (Figure 1). In tissues other than the liver, however, *ITIH1*-*ITIH4* transcripts were only weakly expressed. Importantly, the predominant expression of *ITIH1*-*ITIH4* in liver tissue was also observed in the FANTOM5 and HPA (Human protein atlas) dataset (Supplementary Figures 1, 2). Further analyzing single-cell RNA-seq data from Single Cell Expression Atlas (https://www.ebi.ac.uk/gxa/sc/home) revealed that *ITIH1*-*ITIH4* were exclusively expressed in hepatocyte, but not other cell types in liver tissues (Supplementary Figure 3). By exploiting RNA-sequencing data of over 1,000 cell lines from the Cancer Cell Line Encyclopedia (CCLE) (https://www.broadinstitute.org/ccle), we were able to show that *ITIH1*-*ITIH3* were most strongly expressed in liver cancer cell lines (*ITIH4* expression seems to be silenced across all cancer cell lines) (Supplementary Figure 4). This observation was confirmed by analyzing the RNA-seq data of 64 cell lines from The Human Protein Atlas (HPA) (https://www.proteinatlas.org/): *ITIH1*-*ITIH4* were consistently expressed in Hep G2 (human hepatocellular liver carcinoma) cells, but no other cell lines (Supplementary Figure 5).

### Analysis of ITIH family gene expression levels in tumor and non-tumor tissues

**Figure 2:**
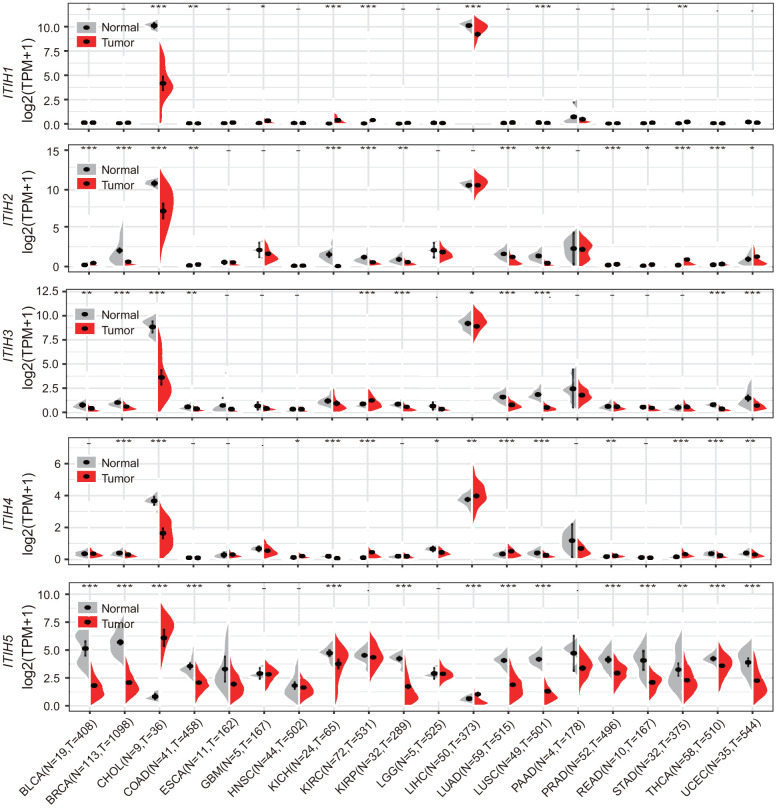
**mRNA expression difference of *ITIHs* between tumor and corresponding normal samples from TCGA database.** Grey, normal control samples; red, tumor samples. *P < 0.05; **P < 0.01; ***P < 0.001; “-“not significant. See Supplementary Figure 6 for supporting data.

The previous pan-cancer study of *ITIH* family has been restricted to a few cancer types (n = 13) and relatively small sample size (241 tumor and 241 matched normal samples) [4]. Draw on the richness of pan-cancer datasets from the TCGA project, we compared *ITIHs* expression between tumor and adjacent normal tissue across 20 cancer types (7900 tumor and 724 normal samples). We found that, apart from *ITIH5*, *ITIH1*-*ITIH4* were all robustly expressed in liver hepatocellular carcinoma (LIHC) and cholangio carcinoma (CHOL), with no or low expression in other cancer types (Figure 2). Nevertheless, *ITIH1*-*ITIH4* were all strongly down-regulated in CHOL, while in LIHC, reduced expression of *ITIH1*, *ITIH3*, and *ITIH5* were observed (Figure 2). Interestingly, *ITIH5* exhibited relatively higher expression levels averaged across all cancer types compared to *ITIH1*-*ITIH4* (Supplementary Figure 6A), whereas in LIHC, it showed the lowest expression level (Figure 2). Accordingly, we found significantly negative correlations between *ITIH5* and the other four genes using the pan-cancer expression data (Supplementary Figure 6B). Also, *ITIHs* expressions were generally found to be reduced in lung cancers, including lung squamous cell carcinoma (LUSC) (for *ITIH1*-*ITHI5*) and lung adenocarcinoma (LUAD) (for *ITIH2*, *ITIH3*, and *ITIH5*); breast cancer (BRCA) (for *ITIH2*-*ITIH5*); colon adenocarcinoma (COAD) (for *ITIH1*, *ITIH3*, and *ITIH5*); and kidney chromophobe (KICH) (for *ITIH2*, *ITIH4*, and *ITIH5*) (Figure 2). This is consistent with a previous study reporting that down-regulation of *ITIHs* were commonly seen in lung, breast, and renal tumors [4]. Up-regulation of *ITIHs* in cancers, however, was relatively uncommon. For example, *ITIH1* and *ITIH4* were more highly expressed in stomach adenocarcinoma (STAD) and kidney renal clear cell carcinoma (KIRC) as compared with the normal tissues. When combining the normal tissue of the GTEx dataset as controls (27 cancer types), we found that the *ITIHs* were significantly dysregulated in almost all cancer types, for which expression reduction was more commonly seen (Supplementary Figure 7). In summary, *ITIHs* shows globally down-regulated patterns across various cancers, suggesting them as potential tumor suppressors in specific cancers.

Using exoRBase [11], we further explored the expression pattern of *ITIHs* in human blood exosomes from the following specimens: normal person (NP), coronary heart disease (CHD), colorectal cancer (CRC), hepatocellular carcinoma (HCC), pancreatic adenocarcinoma (PAAD) and whole blood (WhB). The expression levels of *ITIH1*-*ITIH4* were, as expected, relatively high in the blood of HCC samples; whereas for *ITIH5*, this tissue specificity was not seen (Supplementary Figure 8).

### Expression patterns of ITIHs across different pathologic stages in pan-cancers

**Figure 3:**
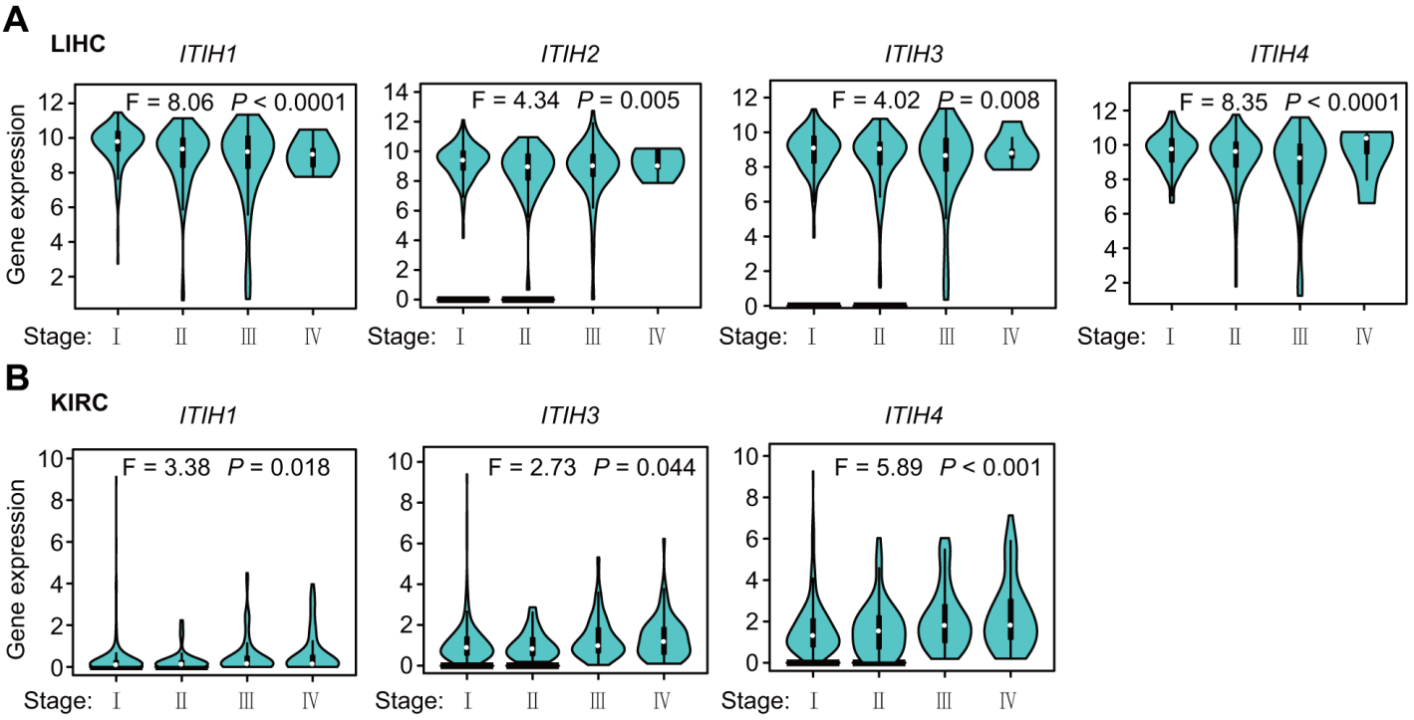
Expression level of *ITIHs* in different pathological stages (stage I, stage II, stage III, and stage IV) of LIHC (**A**) and KIRC (**B**).

Next, we used the “Stage Plot” module of GEPIA2 [12] to investigate whether *ITIHs* expressions might differ between different pathologic stages in pan-cancers. Overall, the expression levels of *ITIHs* were significantly associated with the clinical stage in the following cancers: LIHC (for *ITIH1*-*ITIH4*), KIRC (for *ITIH1*, *ITIH3*, and *ITIH4*), KIRP (for *ITIH2* and *ITIH4*), LUSC (for *ITIH2* and *ITIH4*), STAD (for *ITIH3* and *ITIH5*), PAAD (for *ITIH1*), cervical squamous cell carcinoma (CESC) (for *ITIH4*), ovarian serous cystadenocarcinoma (OV) (for *ITIH4*), adrenocortical carcinoma (ACC) (for *ITIH5*), BRCA (for *ITIH5*), and LUAD (for *ITIH5*) (Figure 3 and Supplementary Figure 9). Noteworthy, we observed a consistent decrease in the expression levels of *ITIH1*-*ITIH4*-especially *ITIH1*-as tumor grade progressed in LIHC (Figure 3A), further highlighting potential tumor-suppressive functions of *ITIH1*-*ITIH4* in this cancer. We also observed that the expression levels of *ITIH1*, *ITIH3*, and *ITIH4* increased with tumor staging of KIRC patients (Figure 3B), as did that of *ITIH2* in KIRP patients (Supplementary Figure 9).

### Prognostic significances of ITIHs in different cancers

**Figure 4:**
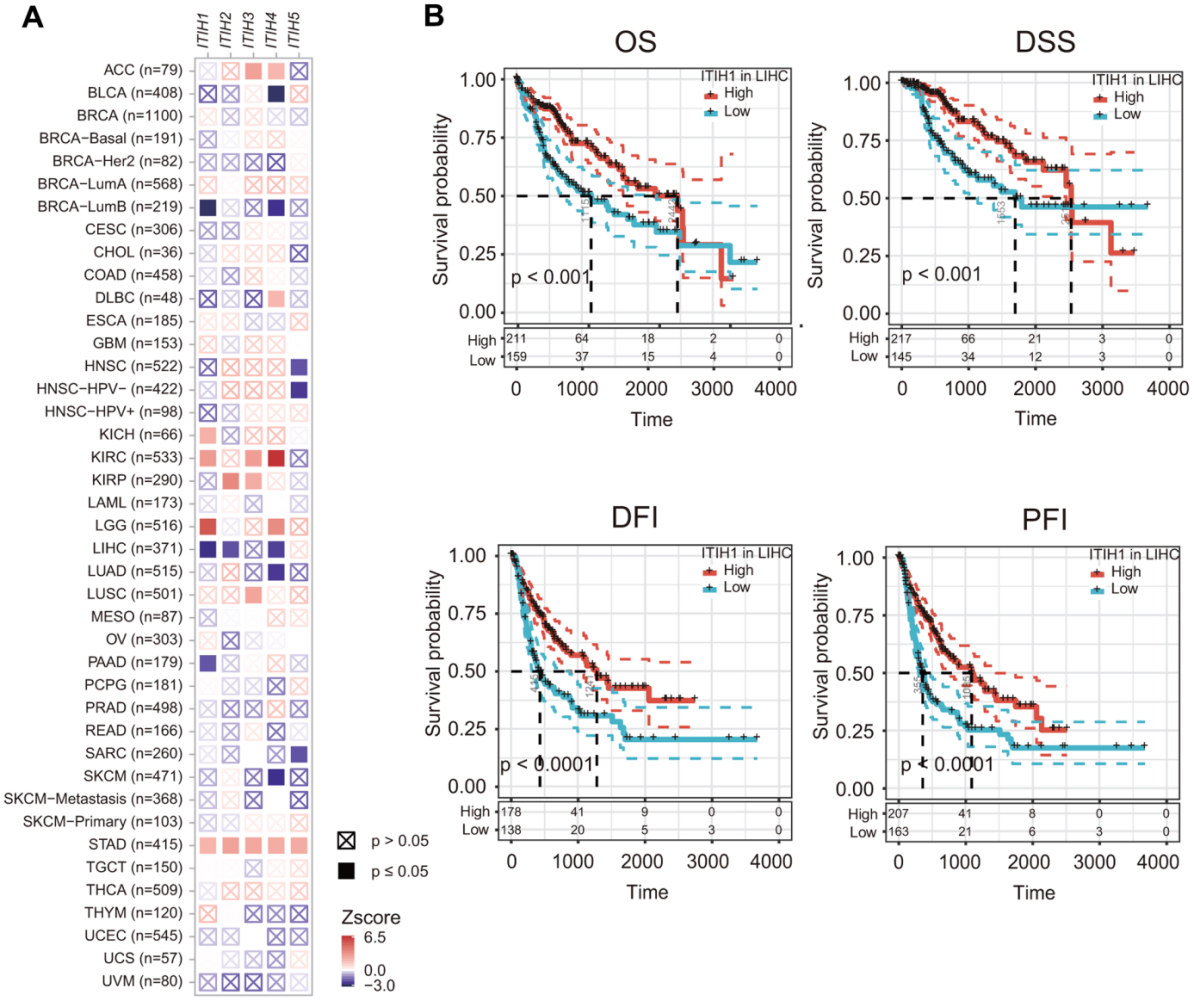
**The prognostic impacts of *ITIHs* in cancers.** (**A**) Association between ITIHs expression and patient prognosis across 33 cancer types as determined by the TIMER2.0 database. (**B**) Kaplan-Meier curves represent OS, DSS, DFI, and PFI of patients with LIHC stratified by the expression levels of *ITIH1*. *ITIH1* expression was significantly associated with OS, DSS, DFI, and PFI in LIHC.

Given that the expression of *ITIHs* were significantly dysregulated in a number of cancers and also related to tumor stage, we asked whether *ITIHs* may have prognostic relevance in cancers. Our analyses based on 33 cancer types revealed that the significance and direction of the associations varied, depending both on the cancer types and genes analyzed (Figure 4A). For example, in STAD and pan-kidney cancers (KIRP, KICH, and KIRC), increased expression of *ITIHs* generally predicted poor overall survival (OS). While in LIHC, a significant beneficial effect on OS was observed for *ITIH1*, *ITIH2*, and *ITIH4* (Figure 4A). Considering the genes queried, *ITIH1* and *ITIH4* were associated with either survival advantage or disadvantage in a number of cancer types, and *ITIH2* and *ITIH5* were only prognostically relevant in a few cancers, but increased expression of *ITIH3* was predominantly associated with worse prognosis (for ACC, KIRC, KIRP, LUSC, and STAD) (Figure 4A).

### Validation of the expression pattern and prognostic significance of ITIH1 in LIHC

**Figure 5:**
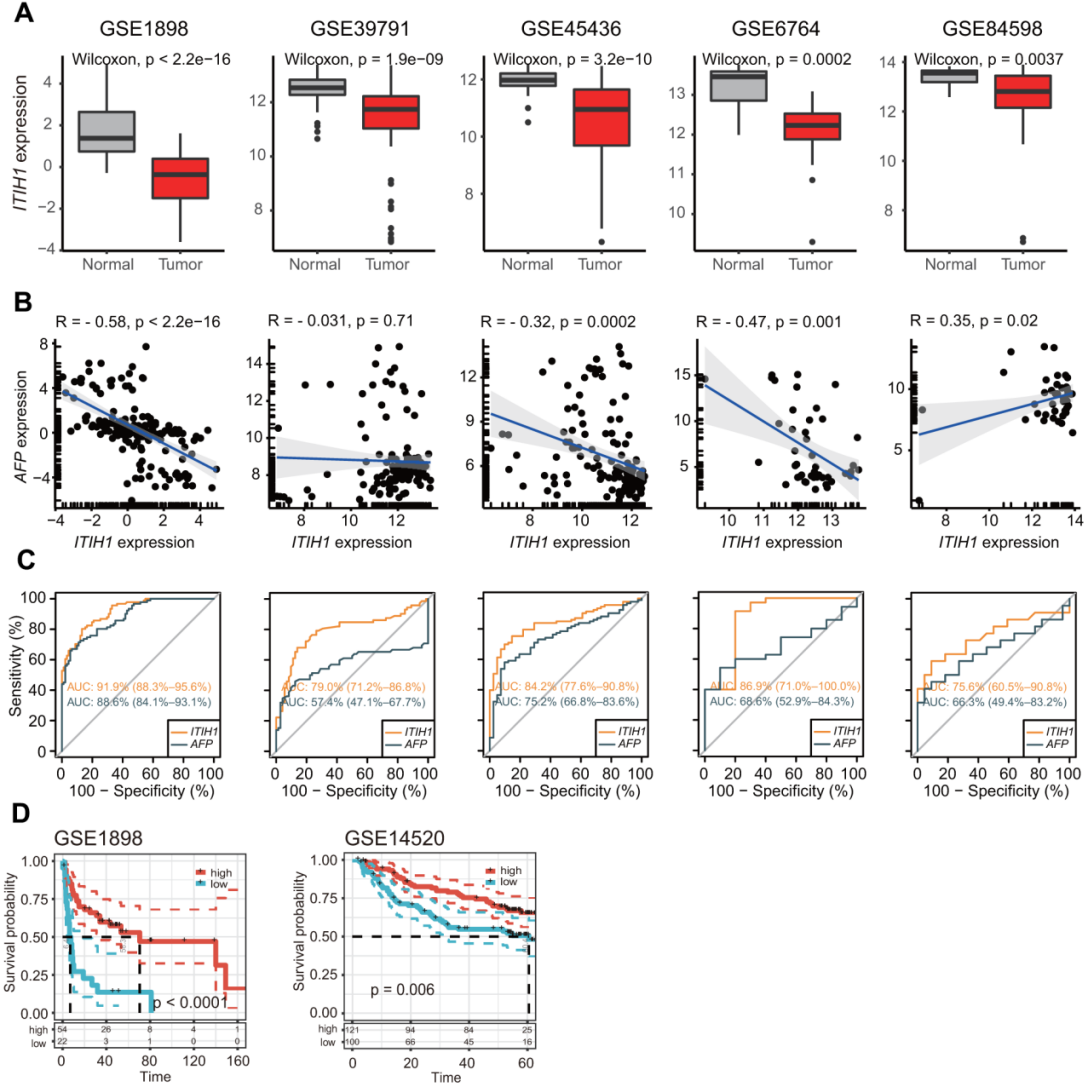
**Independent validation of the differential expression and prognostic significance of *ITIH1* in GEO datasets.** (**A**) Boxplots showing the expression of *ITIH1* in LIHC and normal controls from five GEO datasets (GSE1898, GSE39791, GSE45436, GSE6764, and GSE84598). (**B**) Scatterplots showing the correlation between *ITIH1* and *AFP* expression in the five datasets as described in (**A**). Pearson correlations and p values are indicated. The linear models describing the correlations are depicted as blue lines. The marginal rugs drawn on the axis of the scatter plots were used to show the distributions of two variables. (**C**) Receiver operating characteristic (ROC) curves comparing the diagnostic performances of *ITIH1* (orange curves) with *AFP* (black curves) in the five datasets as described in (**A**). (**D**) Kaplan-Meier curves representing OS of two LIHC cohorts from GEO (GSE1898, n = 76; GSE14520, n = 221) based on *ITIH1* expression.

It is worth mentioning that *ITIH1*, which was significantly down-regulated in LIHC and remarkably decreased with tumor progression, demonstrated the most significant prognostic power for OS (*P* < 0.001) in LIHC compared to the other *ITIH* family members. Importantly, when the other survival endpoints-DSS (disease-specific survival), DFI (disease-free interval), and PFI (progression-free interval)-were analyzed, *ITIH1* appeared to be the only gene that was constantly significant for all endpoints in LIHC (Figure 4B). Moreover, we confirmed the remarkable down-regulation of *ITIH1* in LIHC in five GEO datasets (GSE1898, GSE39791, GSE45436, GSE6764, and GSE84598) (Figure 5A). Using these five datasets, we also analyzed the correlation between the expression of *ITIH1* and alpha-fetoprotein (*AFP*) (the most commonly used diagnostic marker for LIHC). We found that *ITIH1* correlated negatively with *AFP* in three of five datasets, with strongest correlation in the GSE1898 dataset (Figure 5B). Then, the diagnostic performances of the two genes were assessed by analyses of ROC curves. As shown in Figure 5C, the area under the ROC curve (AUC) of *ITIH1* was consistently higher than that of *AFP* in all five datasets analyzed. This suggests that, at the mRNA level, the diagnostic value of *ITIH1* may be at least as good as that of *AFP*, although the utility awaits future experimental validation.

Furthermore, the good prognostic impact of *ITIH1* was validated in two independent cohorts of LIHC patients (GSE1898, n = 76; GSE14520, n = 221) (Figure 5D). Therefore, subsequent analyses will focus on the *ITIH1* gene, especially on its roles in LIHC.

### The genetic and epigenetic features of ITIH1 in pan-cancers

**Figure 6:**
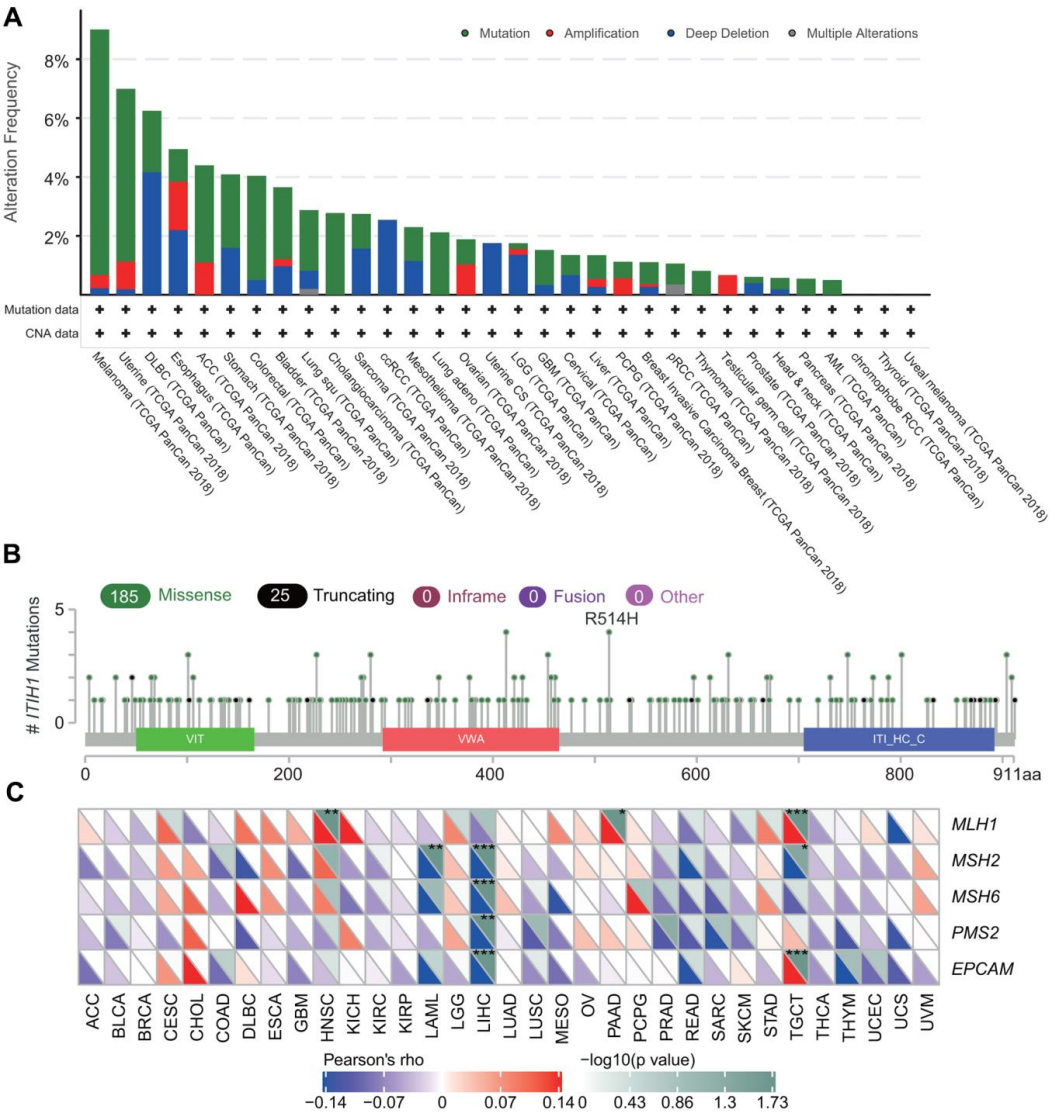
**The genetic features of *ITIH1* in pan-cancers.** (**A**) Genetic alteration frequencies of *ITIH1* across different tumors from TCGA. (**B**) The mutation type and mutation site as determined by cBioportal. (**C**) Correlation between *ITIH1* mRNA expression and mutation levels of five key MMR genes (*MLH1*, *MSH2*, *MSH6*, *PMS2*, *EPCAM*). The lower triangle in each tile indicates coefficients calculated by Pearson’s correlation test, and the upper triangle indicates log10 transformed P-value. *P < 0.05; **P < 0.01; ***P < 0.001.

Next, we explored the genetic alterations of *ITIH1* in TCGA pan-cancer datasets using the cbioportal for Cancer Genomics (http://www.cbioportal.org). We observed that the overall mutation rate of *ITIH1* in cancers is relatively low (less than 10%). Melanoma demonstrated the highest frequency of *ITIH1* mutation (8.33%), followed by uterine cancer (5.86%) (Figure 6A). cBioPortal Oncoprint showed that missense mutation was the main mutation type of *ITIH1* and most mutations were C>T (Supplementary Figure 10). No hot spot mutation site was detected for *ITIH1* in pan-cancers (Figure 6B). For copy number variations (CNVs) of *ITIH1*, amplification was most frequently observed in esophagus cancer (1.65%), while deletion event occurred more often in diffusive large B-cell lymphoma (DLBC) (4.17%) (Figure 6A). In LIHC, despite significantly dysregulated expression of *ITIH1*, the total genetic alteration rate appeared to be very low (1.34%) (Figure 6A).

In addition, we analyzed the correlation between *ITIH1* expression and TMB (Tumor mutational burden)/MSI (Microsatellite instability) across 33 cancer types. We found that *ITIH1* was negatively correlated with TMB of CHOL, head and neck squamous cell carcinoma (HNSC), LUAD, PAAD, rectum adenocarcinoma (READ), STAD, and Thymoma (THYM), but positively correlated with that of Brain lower grade glioma (LGG) (Supplementary Figure 11A). A negative correlation between *ITIH1* expression and MSI was seen in PAAD, Pheochromocytoma and Paraganglioma (PCPG), and STAD, whereas a positive correlation was found for Prostate adenocarcinoma (PRAD) (Supplementary Figure 11B). Interestingly, we found *ITIH1* expression showed significant and negative correlations with mutation levels of four of five key mismatch repair (MMR) genes-*MLH1*, *MSH2*, *MSH6*, *PMS2*, and *EPCAM*/*TACSTD1*-in LIHC (Figure 6C).

**Figure 7:**
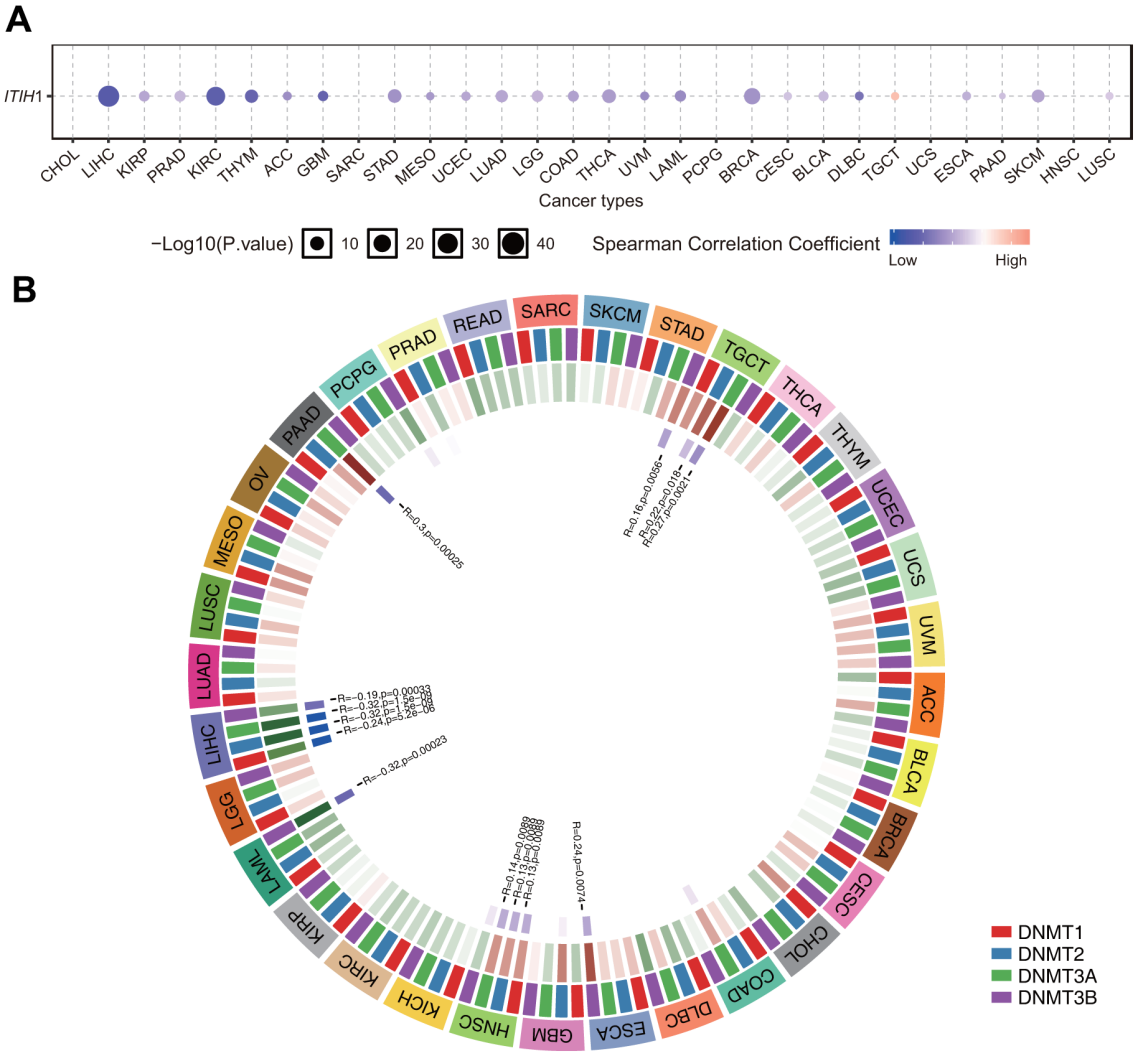
**Relationship between methylation levels and *ITIH1* mRNA expression level in various tumors in TCGA database.** (**A**) Correlation between methylation and *ITIH1* mRNA expression analyzed by the GSCA database. Blue dots indicates negative correlation and red indicates positive correlation. The darker the color, the higher the correlation. The size of the point represents the statistical significance, and the larger the size, the greater the significance. (**B**) Correlation between *ITIH1* expression and the expression levels of four methyltransferases (*DNMT1*: red, *DNMT2*: blue, *DNMT3A*: green, *DNMT3B*: purple).

Epigenetics, especially DNA methylation, also plays a key role in the regulation of gene expression. Using the GSCA database [13], we further examined the correlation between *ITIH1* DNA methylation and expression in pan-cancers. Our results showed that the expression of *ITIH1* was mainly negatively correlated with methylation, with the highest correlation observed in LIHC (Figure 7A). Moreover, we observed significant negative correlations between *ITIH1* expression and the mRNA expression of four DNA-methyltransferases (*DNMT1*, *DNMT2*, *DNMT3A*, and *DNMT3B*) in LIHC, while in other cancers, the correlations were mostly not significant or only significant for less than four DNMT members (Figure 7B). Overall, these results demonstrated that the dysregulation of *ITIH1* expression in LIHC may be partially mediated by DNA methylation.

### Association between ITIH1 expression and immune responses in cancer

**Figure 8:**
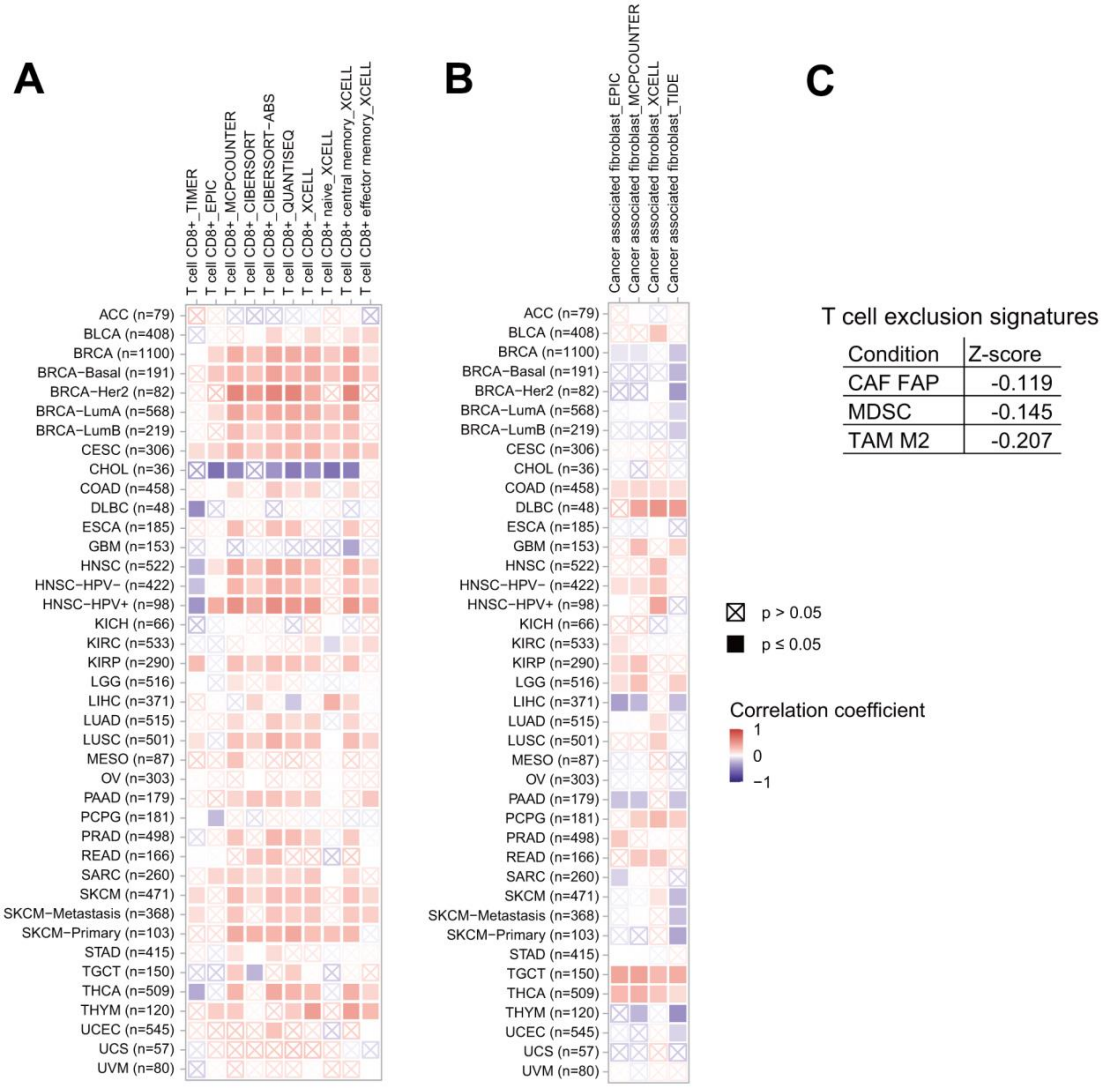
**Association of *ITIH1* expression with tumor microenvironment factors.** Correlation between *ITIH1* expression and immune infiltration of CD8+ T cells (**A**) and cancer-associated fibroblasts (CAFs) (**B**) across different cancers in TCGA. (**C**) Correlation between *ITIH1* expression and T cell exclusion signatures, including FAP+ CAFs, myeloid-derived suppressor cells (MDSC), and tumor-associated M2 macrophages (TAM M2) in the TIDE (Tumor Immune Dysfunction and Exclusion) database.

It is well-known that the immune microenvironment plays key roles both in tumor progression and elimination, therefore it is interesting to analyze the association between *ITIH1* expression and the pro-/anti-tumor immune components. Herein, we used seven algorisms (TIMER, EPIC, MCPCOUNTER, CIBERSORT, CIBERSORT−ABS, QUANTISEQ, and XCELL) to quantify the density of CD8+ T cells in each cancer type, which, were then correlated to *ITIH1* expression levels. We observed an overall positive correlation between the fraction of CD8+ T cells and *ITIH1* expression in pan-cancers except for that of CHOL, where the two components were negatively correlated based on all the algorisms (Figure 8A). Cancer-associated fibroblasts (CAFs) are generally considered to have pro-tumor properties [14]. Our analyses demonstrated that *ITIH1* expression and CAFs abundances were positively correlated in most cancer types (Figure 8B). Noteworthy, a significant negative correlation between *ITIH1* expression and CAFs was observed in LIHC based on 3 of 4 algorisms (EPIC, MCPCOUNTER, XCELL, and TIDE) (Figure 8B). Moreover, using the TIDE (Tumor Immune Dysfunction and Exclusion) database, we found that *ITIH1* expression was also negatively correlated to T cell exclusion signatures, including FAP+ CAFs, myeloid-derived suppressor cells (MDSC), and tumor-associated M2 macrophages (TAM M2) (Figure 8C).

These results led us to further analyze the correlation between *ITIH1* and the expression of several well-known checkpoint genes, since some of which have shown to be promising targets for cancer immunotherapy. We found that the correlation results were not gene-specific but tumor type-specific: *ITIH1* expression did not show correlations with specific checkpoint genes across pan-cancers; however, strong correlations were found between *ITIH1* and most of the checkpoint genes in specific cancer types, such as HNSC, LGG, LIHC, LUSC, mesothelioma (MESO), THYM, and uterine corpus endometrial carcinoma (UCEC) (Supplementary Figure 12). Strikingly, we found that for most cancers *ITIH1* significantly correlated with checkpoint genes in a positive direction except for LIHC in a negative direction (Supplementary Figure 12). In summary, the role of *ITIH1* in LIHC might in favor of immune activation while against immune suppression, further study is needed to test this hypothesis.

### Genes co-expressed with ITIH1 were mainly associated with metabolic pathways

To further assess the role of *ITIH1* in tumors, we derived genes that were significantly co-expressed with it across pan-cancers (r > 0.4, Supplementary data 1). Among the 462 genes were, as expected, *ITIH* family members *ITIH2*, *ITIH3*, and *ITIH4*, with *ITIH4* the most significantly correlated. In addition, some tumor suppressors were identified, such as: *ACY1*, *CDO1*, *CEBPA*, *GLS2*, *MST1*, and *NR0B2*. The prominent feature of the signature associated with *ITIH1* expression was the identification of critical negative regulators for LIHC glycolysis, including *CYP2A6*, *CYP3A4*, *HSD17B13*, *LECT2*, *SLC10A1*, and *SPP2*; notably, high expression of *CYP3A4*, *HSD17B13*, *LECT2*, *SLC10A1*, and *SPP2* were associated with favorable outcomes in LIHC patients [15]. Also noteworthy was the strong enrichment of genes from the solute carrier family (eg, *SLC10A1*, *SLC13A5*, *SLC17A2*, *SLC22A1*, and *SLC22A10*), the Cytochrome P450 (CYP) family (eg, *CYP2A6*, *CYP2B6*, *CYP2C18*, and *CYP2C8*), and the serine protease inhibitor superfamily (eg, *SERPINA10*, *SERPINA4*, and *SERPINA7*).

**Figure 9:**
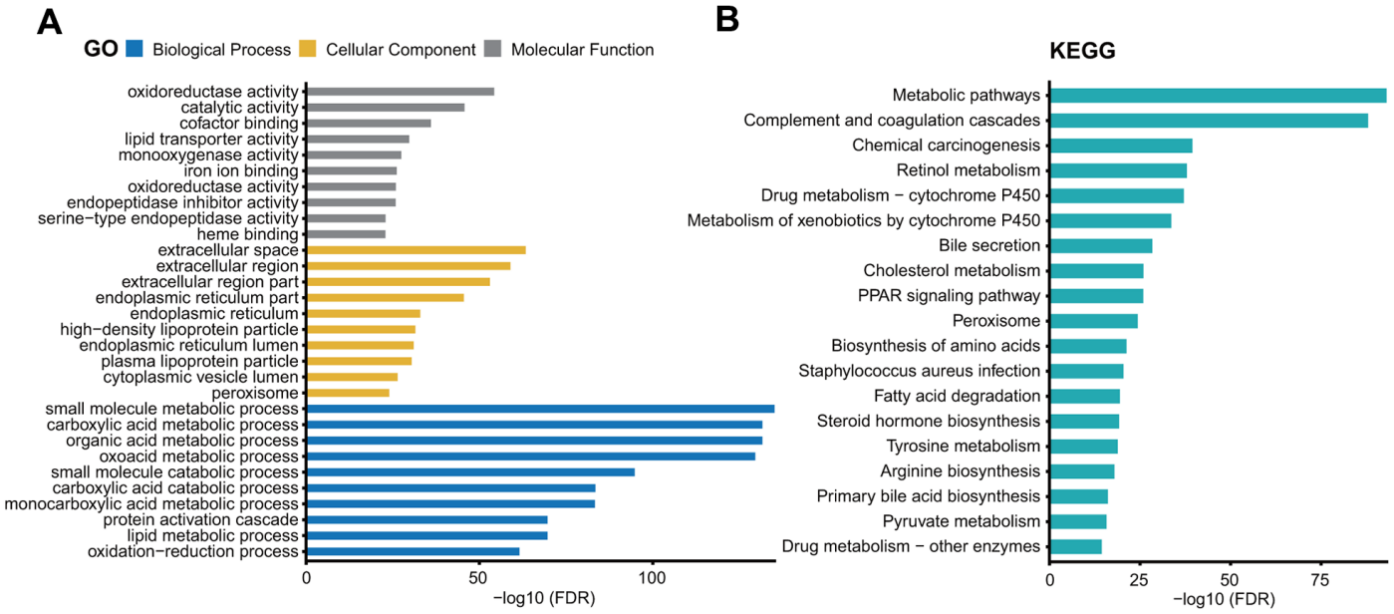
GO (**A**) and KEGG pathway (**B**) analysis of the genes co-expressed with *ITIH1*.

We then performed gene ontology (GO) and Kyoto encyclopedia of Genes and Genomes (KEGG) pathway analysis of these genes using the STRING database [16]. We found that the genes co-expressed with *ITIH1* were mostly involved in the metabolic and catabolic biological processes. Another highly overrepresented GO term was the extracellular region. The detailed results were shown in Figure 9A. The KEGG pathway analysis revealed that most of the terms were related to metabolic pathways; included among these were retinol metabolism, drug metabolism- cytochrome P450, metabolism of xenobiotics by cytochrome P450, cholesterol metabolism, tyrosine metabolism, pyruvate metabolism, and drug metabolism-other enzymes (Figure 9B).

## DISCUSSION

The *ITIH* proteins, on the basis of their protease inhibitor nature, have long been postulated to be involved in the stabilization of extracellular matrices [5]. It has also been shown that two members-*ITIH1* and *ITIH3*-could have anti-metastasis effects [7]. Since a critical process during cancer invasion and metastasization was the remodeling of the extracellular matrix (ECM) [6], one can reasonably hypothesize that *ITIHs* may act as vital tumor suppressors in cancer development. However, only a limited number of studies have explored the expression and significance of *ITIHs* in cancers. Although an early study has investigated the expression patterns of *ITIHs* in a few cancer entities and normal human tissues [4], their analyses have been restricted to limited cancer types and relatively small sample sizes, and the link between *ITIH* members and patient outcome is largely undetermined. Our study was therefore mainly aimed to validate and extend their findings. We first analyzed the expression levels of *ITIHs* in normal tissues in the GTEx, HPA and FANTOM5 databases. We observed that *ITIH1*-*ITIH4* were predominantly expressed in the liver in all three databases, consistent with the previous finding [4]. This may be because the polypeptide precursors of *ITIH1*-*ITIH3* were synthesized primarily in the liver [1], while *ITIH4* has also been shown to play important roles in liver development and regeneration [17]. *ITIH5*, on the contrary, demonstrated the lowest expression in the liver and it showed a significantly negative correlation with *ITIH1*-*ITIH4* in pan-cancer datasets. This indicates *ITIH5* may have distinct expression pattern and functional role with that of *ITIH1*-*ITIH4*, future studies will be necessary to decipher the specific function of these genes in different tissues and the mechanisms through which they are differentially distributed.

The previous pan-cancer study has reported that *ITIHs* were mostly down-regulated in cancers, such as breast, lung, and colon cancers [4]. Our in-silico analyses using the TCGA pan-cancer datasets showed that, despite that *ITIH1*-*ITIH4* were significantly altered in several cancer types, their basal expression levels in most cancers and corresponding normal tissues were extremely low, except for CHOL and LIHC. We deemed that a gene with tumor-suppressive functions that are suppressed during tumorigenesis should at least be expressed in the corresponding normal tissue. Therefore, some of the differences may be observed by chance. Prospective clinical studies are needed to validate these results. It is noteworthy that *ITIH1*, which was highly expressed in the liver, appeared as the most significantly down-regulated member in LIHC among all *ITIHs*; the remarkable down-regulation was also observed in five independent LIHC datasets from GEO. Strikingly, ROC curve analyses identified *ITIH1* with a strong discriminatory potential between LIHC and normal controls, even superior to that of *AFP*. These findings provide strong evidence for a novel tumor suppressor function of *ITIH1* in liver cancer. Moreover, we observed a consistent decrease of *ITIH1* expression as LIHC progressed from early to advanced stages. Although the expression levels of *ITIH2*, *ITIH3*, and *ITIH4* also differed in different tumor stages of LIHC, the expression change directions were not always identical. A previous study has demonstrated *ITIH4* as a prospective diagnostic marker in HCC that outperformed the commonly used AFP; they found that *ITIH4* was declining during the progression of LIHC [9], which was partially consistent with our findings. Taken together, we reasoned that *ITIH1* would be at least equally suitable for diagnostic purposes in LIHC as *ITIH4*. Nevertheless, our findings were entirely based on mRNA levels reported in the TCGA study, other approaches, such as immunohistochemistry (IHC) and western blotting, are recommended for validating *ITIH1* expression at the protein level.

Another major limitation of the previous study was that they have only briefly investigated the prognostic significance of *ITIH2* in breast cancer, in which *ITIH2* was neither associated with overall survival (OS) nor recurrence-free survival (RFS) [4]. Our analyses, in contrast, provide a comprehensive view of the prognostic landscape of *ITIH* members across human cancers. We found the *ITIH* genes had a mixed association with clinical outcome (both advantage and disadvantage) that is dependent on the cancer type tested and the genes queried. However, we do note that *ITIHs* were generally associated with a survival advantage in LIHC. Notably, further analyses revealed *ITIH1* as the only member that was significantly associated with all survival endpoints, including OS, DSS, DFI, and PFI, and its predictive value for OS was validated in two independent LIHC cohorts. Overall, these results suggest *ITIH1* as a novel prognostic indicator in LIHC, which is definitely worth further investigation.

We then tested the genetic alteration of *ITIH1* in cancers. Our results showed that the mutation frequencies of *ITIH1* in cancers appeared to be quite low, and the main mutation type was missense mutation. In addition, we found the methylation level of *ITIH1* was significantly negatively correlated with its expression level in LIHC. The data indicates that dysregulated expression of *ITIH1* may be influenced by promoter methylation in LIHC, but was unlikely to be regulated by its mutation status. Further studies should be conducted to determine the explicit regulatory mechanisms.

Given that *ITIH1* was significantly down-regulated in LIHC and closely correlated with both tumor grade and patient outcome, we decided to determine its potential functional role in cancers. Through GO and KEGG analysis, we observed that one highly enriched ontology was the extracellular region, which is unsurprising since the *ITIHs* were primarily found in the extracellular matrices of various organs [8]. Interestingly, the most over-represented terms of GO and KEGG pathway analyses turned out to be the metabolic process. For example, members of the Cytochrome P450 (CYP) family, which is largely responsible for the metabolism of cancer drugs, were co-expressed with *ITIH1*. Also noteworthy was the enrichment of critical negative regulators for LIHC glycolysis as reported by a recent study [15]. The field of cancer metabolism has recently been revived with a renewed interest in a phenomenon termed anaerobic glycolysis, which was known to occur during tumor progression and profoundly contributes to the aggressive phenotypes of cancer cells. Therefore, it will be of great interest to determine the functional relationship between *ITIH1* expression and cancer glycolysis metabolism in LIHC.

This study has certain limitations: all the analyses were performed based on the expression of *ITIHs* at the mRNA level, and the conclusions were deduced from bioinformatics analyses, lacking any rigorous mechanistic interpretation from supporting experimental data. Therefore, further research is needed to validate our results and to investigate the biological functions of *ITIH1* in LIHC. That said, the large sample size and independent validation of our findings would still make the main conclusions reliable and generalizable.

In summary, our study confirmed the expression pattern of *ITIHs* reported by a previous pan-cancer analysis, but in a broader view rather than in a limited number of cancer types. We also extended their findings by investigating the prognostic value of *ITIHs* across pan-cancers. Importantly, we for the first time recognized *ITIIH1* as a novel tumor-suppressor gene in LIHC. Our results showed that *ITIH1* was significantly down-regulated in LIHC, and its expression was closely related to tumor stage and survival. Finally, our findings shed light on the functional role of *ITIH1* in cancers, suggesting a strong correlation between *ITIH1* expression and metabolic pathways.

## MATERIALS AND METHODS

### Analysis of gene expression data

First, the mRNA expression data of *ITIH* family in normal tissues were obtained from the Genotype-Tissue Expression (GTEx) project [18]. To confirm the expression patterns of *ITIHs* in normal tissues, we then consulted the HPA (Human protein atlas) and FANTOM5 dataset from the human protein atlas database (http://www.proteinatlas.org/) [19]. Transcripts of *ITIHs* across different cell types in the liver tissue were visualized using Single Cell Expression Atlas (https://www.ebi.ac.uk/gxa/sc/home). Expression data of *ITIHs* for over 1000 cancer cell lines were accessed from Cancer Cell Line Encyclopedia (CCLE) (https://www.broadinstitute.org/ccle) [20]. RNA-seq data of 64 cell lines from The Human Protein Atlas (HPA) ((https://www.proteinatlas.org/) [19] were utilized to validate expression patterns of *ITIHs* in different cancer cell lines.

To explore the expression differences of *ITIHs* between tumor and the corresponding normal tissues across different cancer types, we analyzed TCGA RNA-seq data of 20 cancer types with matched normal samples (7900 tumor and 724 normal). Then, expression data from GTEx were combined with TCGA data, in order to extend the analyses to more cancer types and enlarged samples sizes. The expression levels of *ITIHs* in human blood exosomes were obtained from exoRBase (http://www.exorbase.org/) [11]. In addition, we explored the expression levels of *ITIHs* in different pathologic stages across pan-cancers using the “Stage Plot” module of GEPIA2 web server (http://gepia2.cancer-pku.cn/#analysis) [12]. To validate the differential expression of *ITIH1* between LIHC and normal tissue, we further retrieved five datasets from Gene Expression Omnibus (GEO) (https://www.ncbi.nlm.nih.gov/geo/) under accession number GSE1898, GSE39791, GSE45436, GSE6764, and GSE84598.

### Survival analysis

We used the “Gene Outcome” module of TIMER2.0 (http://timer.cistrome.org/) [21] to analyze the association between *ITIHs* expression and clinical outcomes across 33 cancer types. The association between transcript levels of each member of *ITIH* family and overall survival (OS) across different cancers were tested in univariate Cox regression models. Specifically, LIHC patients were divided into those with high and low *ITIH1* expression, according to the optimal cut-off determined by the X-tile method [22]. We then performed Kaplan-Meier analysis (log-rank test) to compare the survival differences of two groups regarding the following survival endpoints: OS, disease-specific survival (DSS), disease-free interval (DFI), and progression-free interval (PFI). To further confirm the prognostic value of *ITIH1* in LIHC, two GEO datasets (GSE1898 and GSE14520) with available survival information/outcome data were utilized.

### Genetic and epigenetic alteration analysis

The genetic alterations of *ITIH1* in pan-cancers, including somatic mutations, amplification, and deep deletion were assessed through the cbioportal for Cancer Genomics (http://www.cbioportal.org) [23]. Briefly, we first queried “*ITIH1*” after selecting “TCGA Pan-Cancer Atlas Studies” using this web portal. Then, genetic alteration frequencies across TCGA pan-cancer studies were visualized via the “Cancer Types Summary” module. Oncoprint of *ITIH1* mutations in various tumors was drawn through the “OncoPrint” module and the mutated site information of *ITIH1* was displayed via the “Mutations” module. Finally, the GSCALite (http://bioinfo.life.hust.edu.cn/web/GSCALite/) web server [13] was used to analyze the correlation between *ITIH1* expression and methylation in TCGA pan-cancer datasets.

### Immune infiltration analysis

We used the “Gene” module of TIMER2.0 (http://timer.cistrome.org/) [21] to explore the association between gene expression and immune cell infiltration/abundances in TCGA datasets. For our purposes, only CD8+ T cells and cancer-associated fibroblasts (CAFs) were selected for analysis. The immune infiltration levels were estimated by algorithms including TIMER, EPIC, MCPCOUNTER, CIBERSORT, CIBERSORT−ABS, QUANTISEQ, and XCELL. The correlation results were visualized as heatmaps. The TIDE (Tumor Immune Dysfunction and Exclusion) database was used to analyze the relationship between *ITIH1* expression and three T cell exclusion signatures-that is-FAP+ CAFs, myeloid-derived suppressor cells (MDSC), and tumor-associated M2 macrophages (TAM M2).

### Co-expression analysis and functional enrichment analysis

We used the “Similar Gene Detection” module of GEPIA2 [12] to derive genes that were co-expressed with *ITIH1* based on TCGA pan-cancer datasets, and the genes with Pearson correlation coefficients more than 0.4 were considered most related to *ITIH1*. Next, we performed Gene Ontology (GO) analysis and Kyoto Encyclopedia of Genes and Genomes (KEGG) pathway analysis of *ITIH1*-related genes using the STRING database (http://www.string-db.org/) [16]. GO and KEGG terms with false discovery rate (FDR)-corrected p values less than 0.05 were considered as significantly enriched. For displaying purposes, the top 10 GO terms of each three GO domains—biological process (BP), cellular component (CC), and molecular function (MF), and the top 20 KEGG pathway terms were visualized as bar plots.

### Statistical analysis

Wilcoxon rank sum tests were used to compare differences between two groups and one-way ANOVA was used for differences among at least 3 groups. Correlation between two continuous variables was determined by Pearson’s or Spearman’s rank correlation test. We used the SangerBox tool (http://sangerbox.com/) to investigate the correlation between *ITIH1* expression and tumor mutational burden (TMB), microsatellite instability (MSI), mutation levels of mismatch repair (MMR) genes, and expression levels of DNA-methyltransferases and checkpoint genes across various cancers from the TCGA project. All statistical analyses and visualizations were performed using either indicated web servers or R version 3.5.3. Specifically, the “gganatogram” package [24] was used to display *ITIHs* expression on anatograms, “ggplot2” and “ggpubr” for visualization of box, scatter and bar plots, “pROC” for generating Receiver operating characteristic (ROC) curves, and “survminer” for plotting survival curves. All statistical tests were two-sided with p-values less than 0.05 considered significant.

## Supplementary Material

Supplementary Figures

Supplementary Data 1
