# Supplementary material for: Pan-cancer analysis identifies ITIH1 as a novel prognostic indicator for hepatocellular carcinoma
Source: Aging (Albany NY). 2021 Mar 21;13(8):11096–119. doi: 10.18632/aging.202765 (PMC8109120; doi:10.18632/aging.202765)
Supplement: Supplementary Figures [file aging-13-202765-s001.pdf]

SUPPLEMENTARY FIGURES

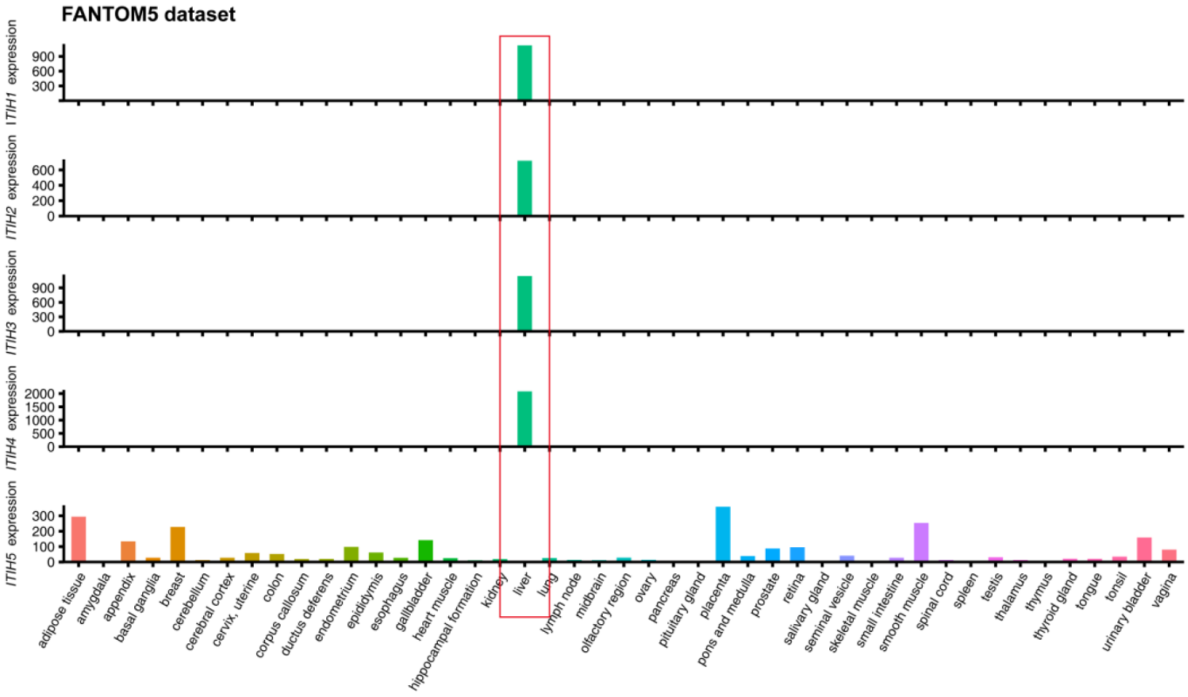

Supplementary Figure 1. mRNA expression levels of ITIHs in normal tissues in the FANTOM5 dataset.

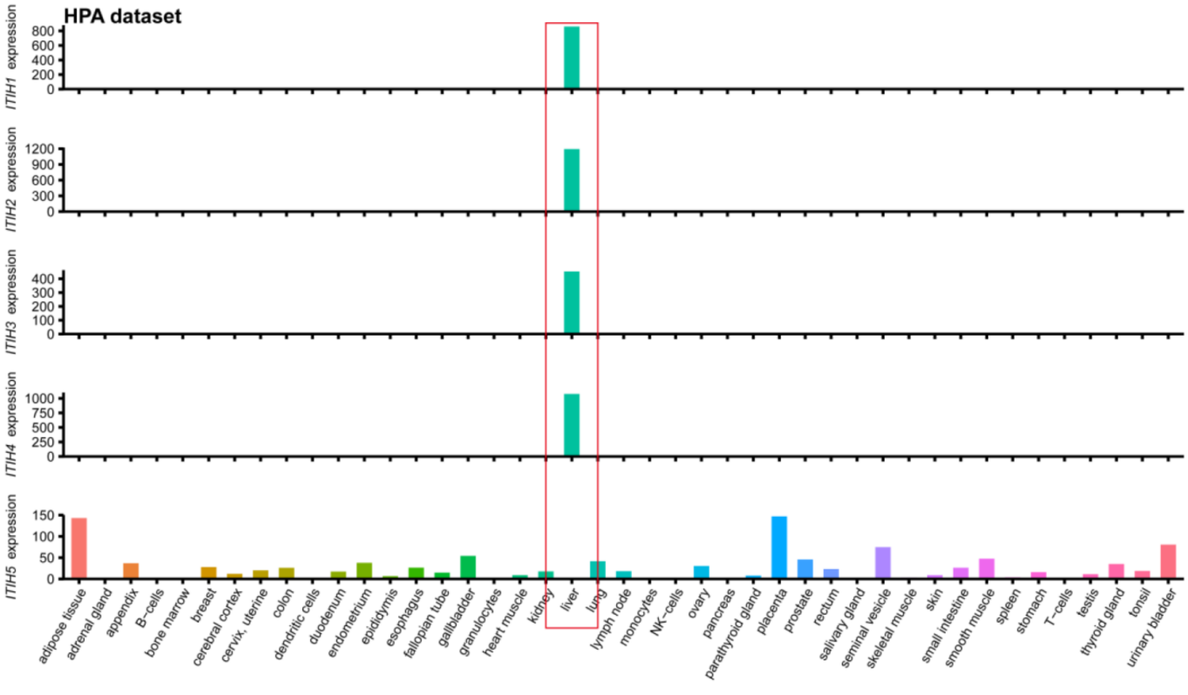

Supplementary Figure 2. mRNA expression levels of ITIHs in normal tissues in the HPA (Human protein atlas) dataset.

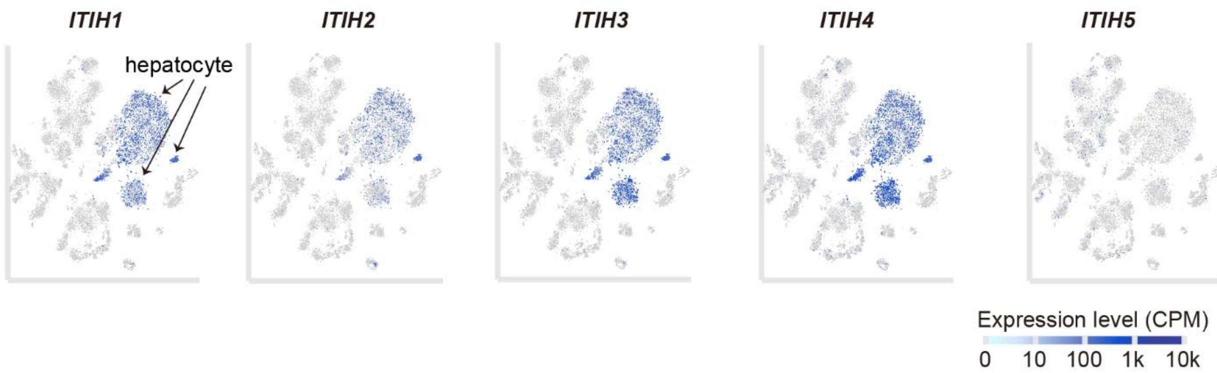

Supplementary Figure 3. mRNA expression levels of *ITIHs* in single cells of the liver tissue via the Single Cell Expression Atlas (https://www.ebi.ac.uk/gxa/sc/home).

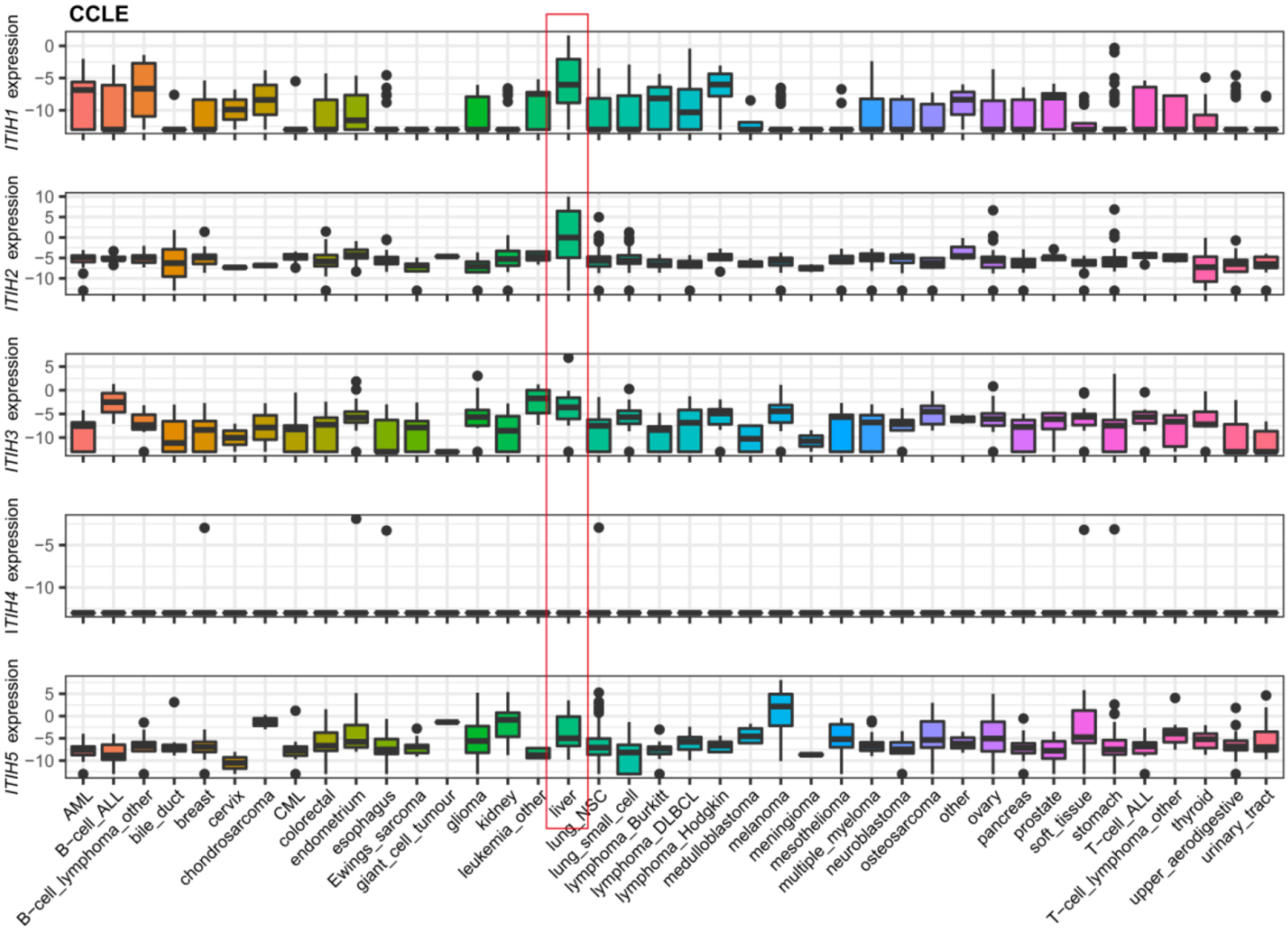

Supplementary Figure 4. mRNA expression levels of *ITIHs* in various tumor cell lines from the Cancer Cell Line Encyclopedia (CCLE) database.

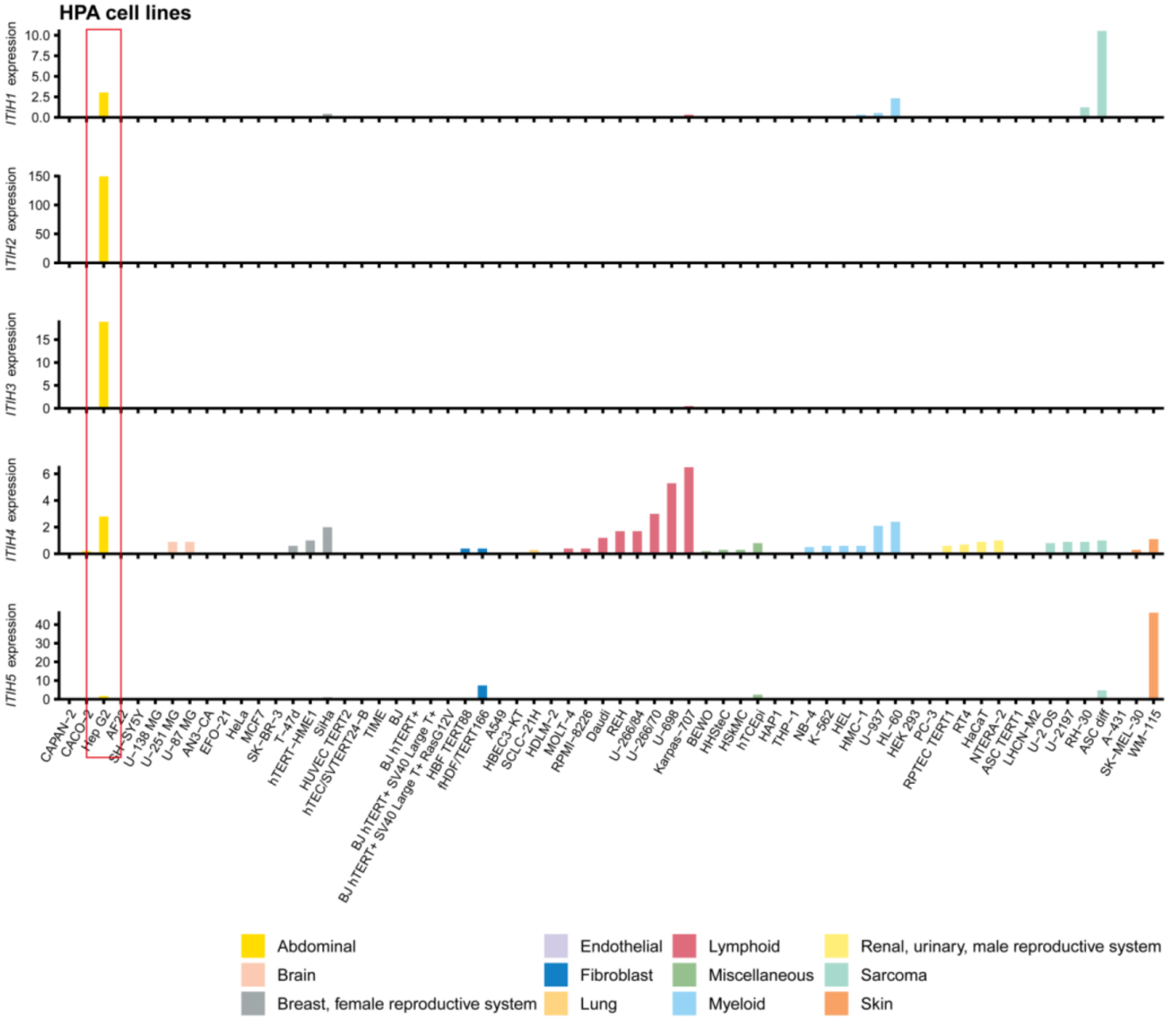

Supplementary Figure 5. mRNA expression levels of *ITIHs* in various tumor cell lines from the Human Protein Atlas (HPA) database.

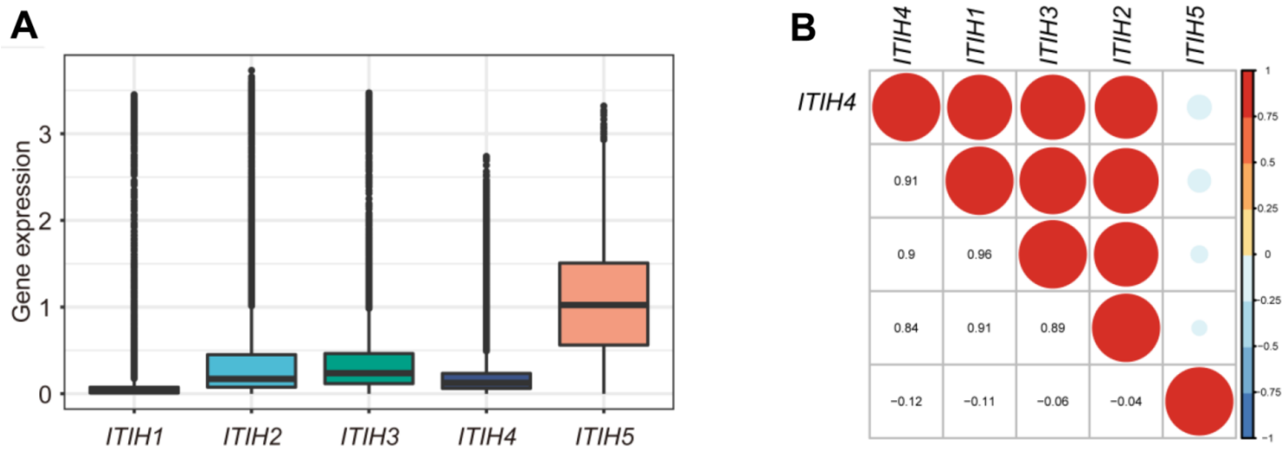

Supplementary Figure 6. The expression pattern of *ITIHs* in TCGA pan-cancer expression data. (**A**) mRNA expression levels of *ITIHs* averaged across all TCGA cancer types. (**B**) Correlation matrix of the expression of ITIH family members in TCGA pan-cancer expression data.

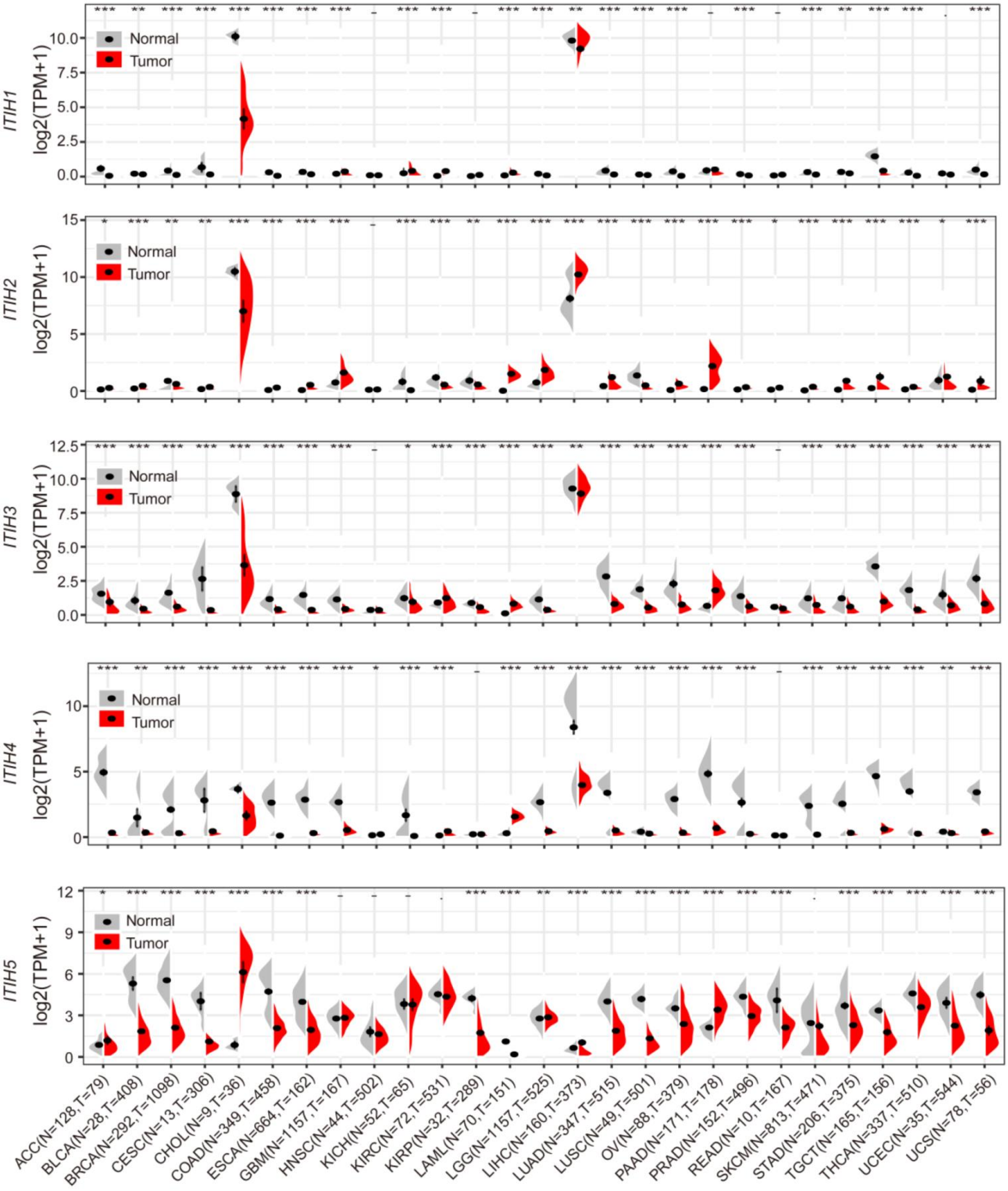

Supplementary Figure 7. mRNA expression difference of *ITIHs* between tumor and normal samples, combining data from TCGA and GTEx databases. Grey, normal control samples; red, tumor samples. **P* < 0.05; ***P* < 0.01; ****P* < 0.001; -not significant.

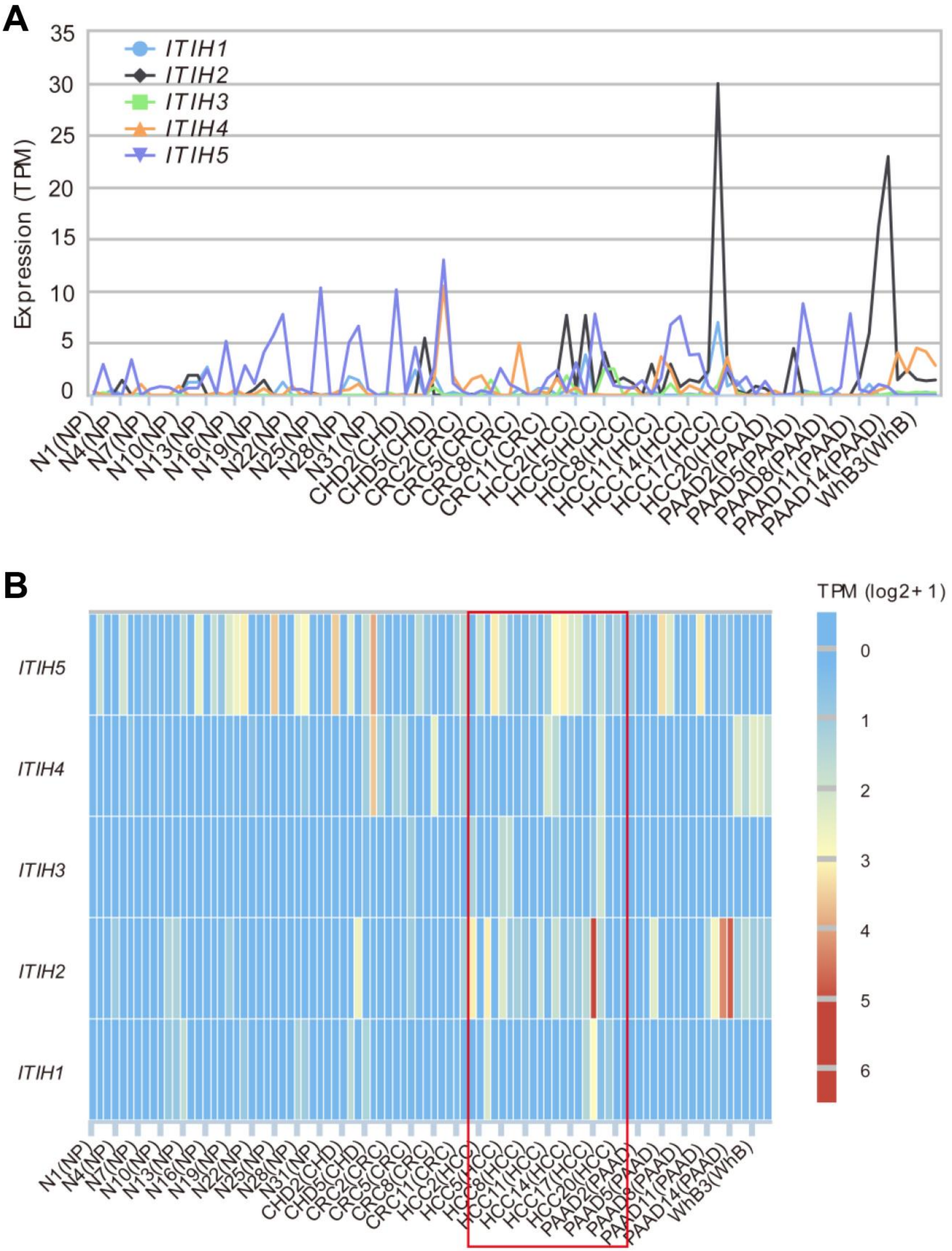

Supplementary Figure 8. mRNA expression levels of *ITIHs* in human blood exosomes of different specimens using exoRBase. The expression patterns were displayed as line chart (**A**) and heat map (**B**), respectively.

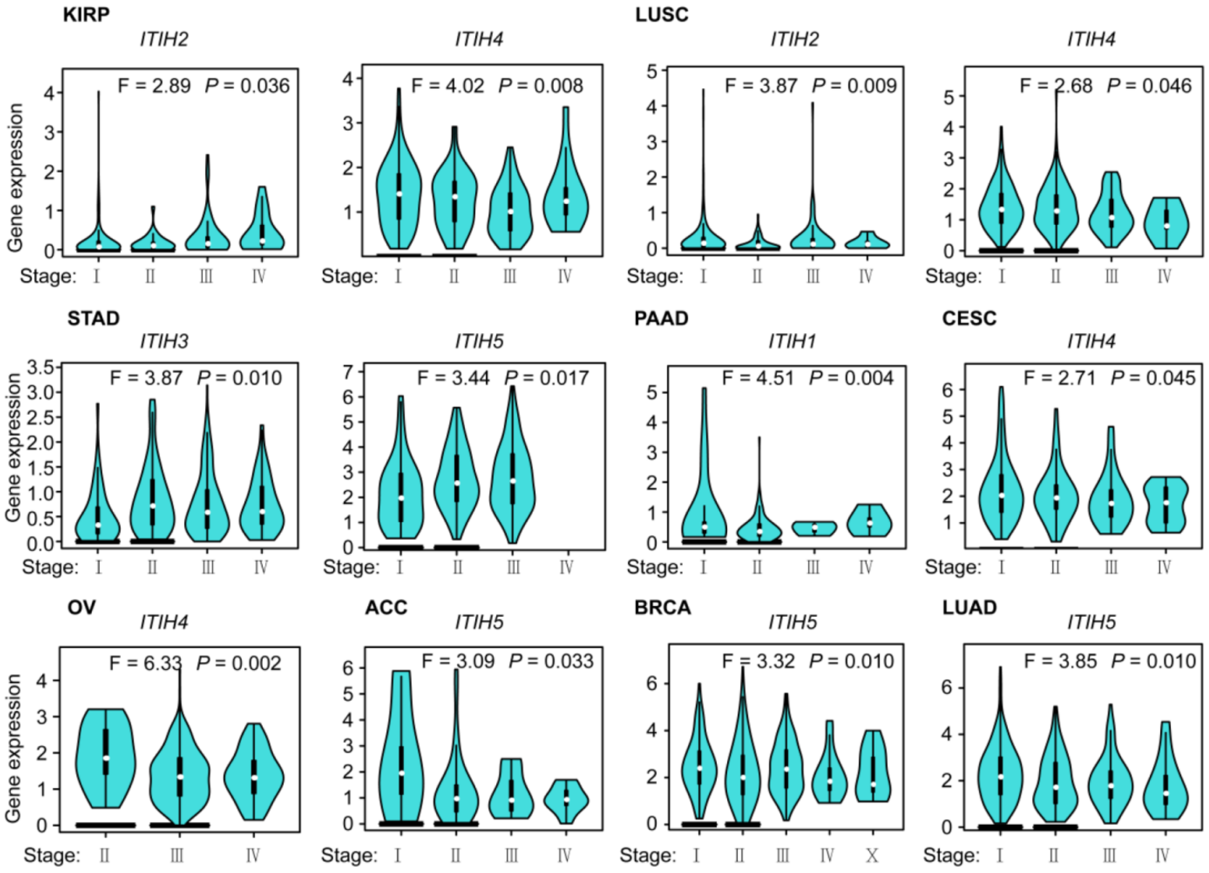

Supplementary Figure 9. Expression level of *ITIHs* in different pathological stages of KIRP, LUSC, STAD, PAAD, CESC, OV, ACC, BRCA, and LUAD.

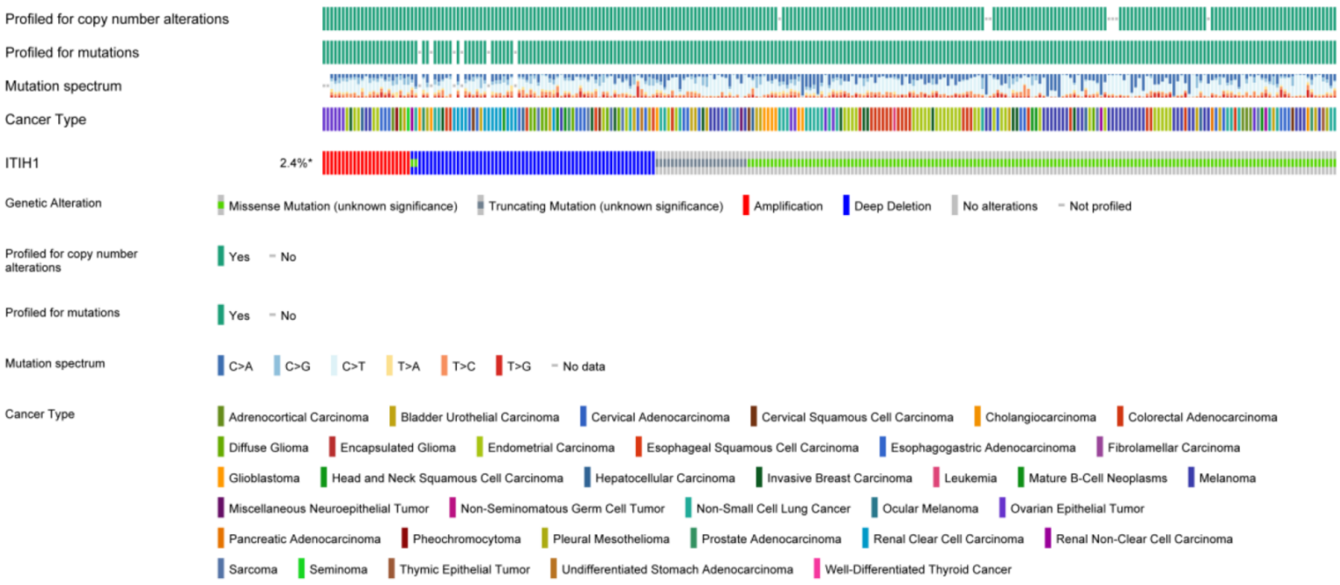

Supplementary Figure 10. cBioPortal Oncoprint showing the mutation spectrum of *ITIH1* across TCGA pan-cancer studies. Each vertical bar represents a patient.

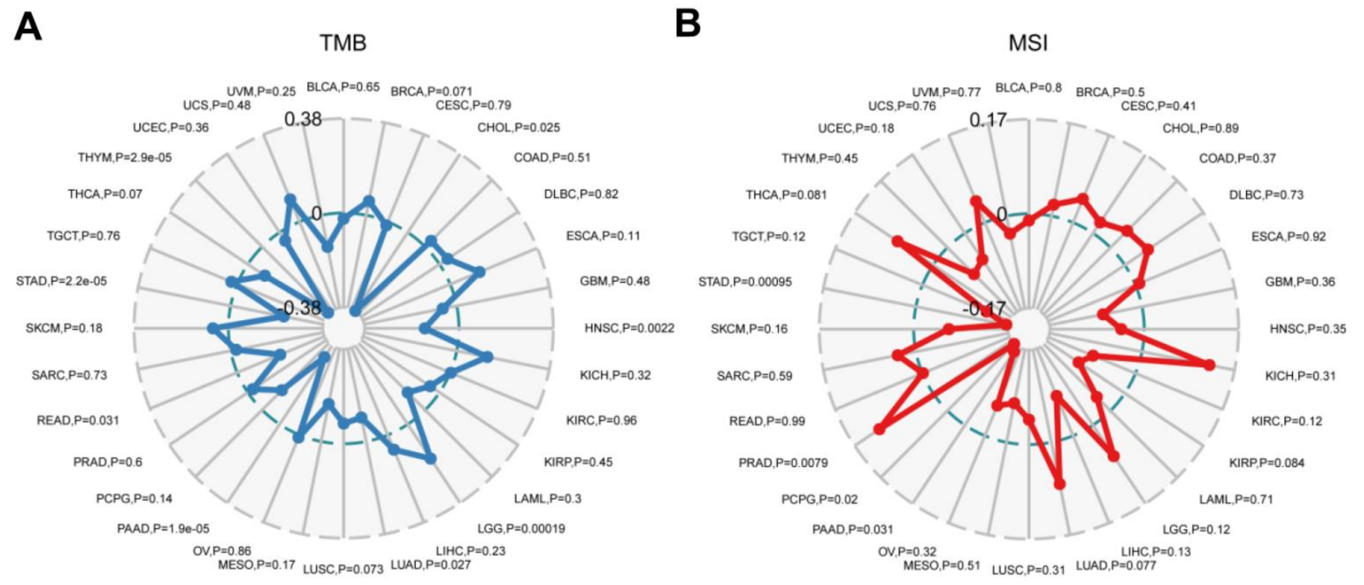

Supplementary Figure 11. Correlation between TMB (**A**), MSI (**B**) and *ITIH1* mRNA expression levels in various cancers in the TCGA database.

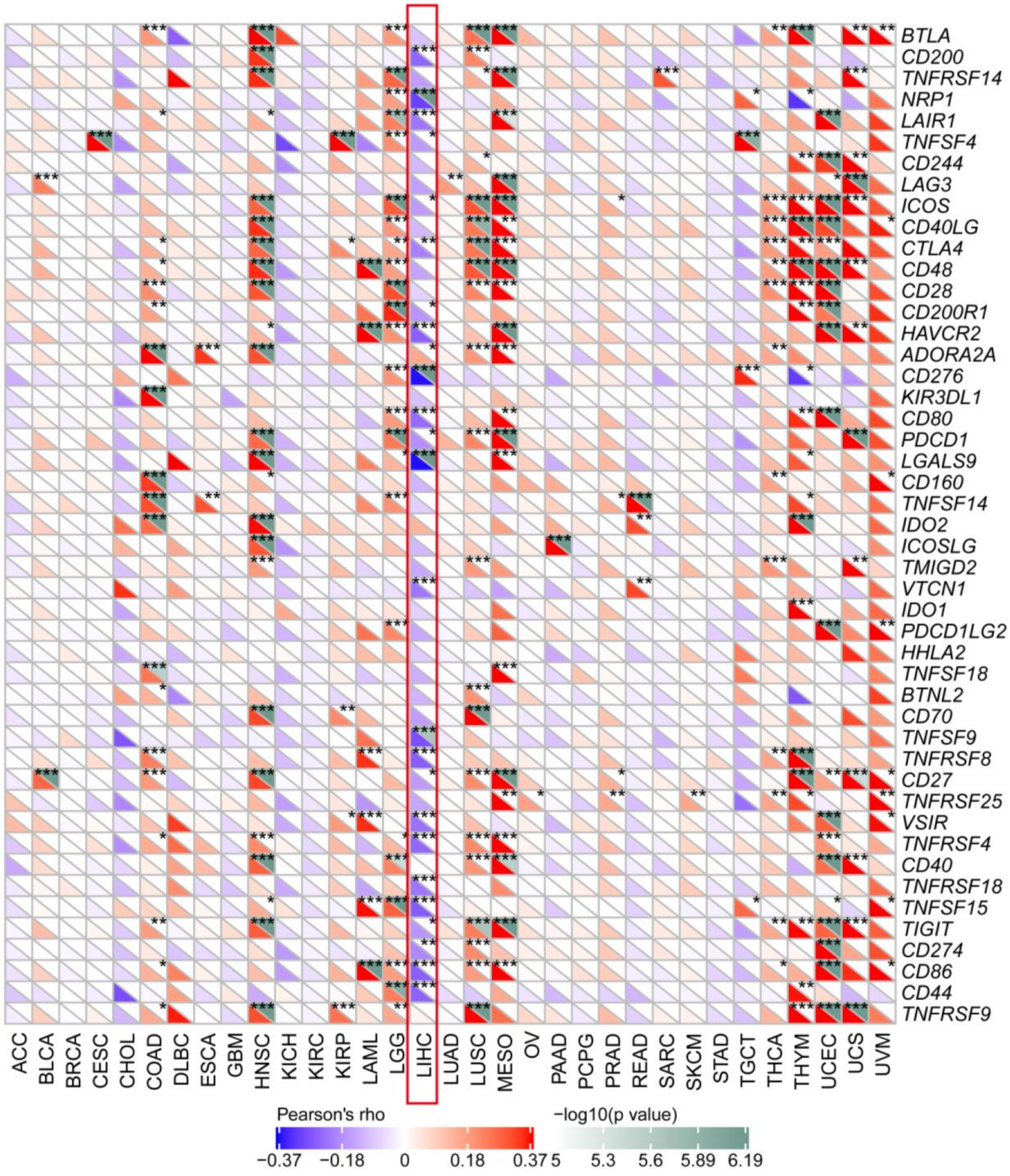

Supplementary Figure 12. Correlation between *ITIH1* mRNA expression and the expression levels of key immune checkpoints in different cancers from the TCGA database.
